# Adipose Tissue Formation Utilizing Fat Flap Distraction Technique

**DOI:** 10.1038/s41598-017-05547-y

**Published:** 2017-07-12

**Authors:** Myung Chul Lee, Won Jai Lee, Byung Il Lee, Kee Yang Chung, Jae Woo Kim, Eun Hye Kang, Yong Oock Kim

**Affiliations:** 1 Department of Plastic and Reconstructive Surgery, Konkuk University School of Medicine, Seoul, Korea; 2 Department of Medicine, Yonsei University Graduate School of Medicine, Seoul, Korea; 3 Department of Plastic and Reconstructive Surgery, Institute for Human Tissue Restoration, Yonsei University College of Medicine, Seoul, Korea; 4 Department of Plastic and Reconstructive Surgery, Korea University College of Medicine, Seoul, Korea; 5 Department of Dermatology, Cutaneous Biology Research Institute, Yonsei University College of Medicine, Seoul, Korea; 6 Department of Biochemistry and Molecular Biology, Integrated Genomic Research Center for Metabolic Regulation, Institute of Genetic Science, Yonsei University College of Medicine, Seoul, Korea

## Abstract

Co-regulation between adipocytes and supporting vasculature is considered an important process in adipose tissue generation. The objective of this study was to evaluate the mechanical and biological effects of a distraction technique on adipose tissue formation and maintenance. Based on the hypothesis that fat flaps gradually receding from each other can develop an adipose tissue construct, perforated polycarbonate syringe-shaped chambers were implanted in a rabbit model. Latency (1 week) and distraction (3 weeks) periods were followed by a consolidation period in the experimental groups (4, 8, and 12 weeks). In the distraction group, the volume of fat pad gradually increased up to 16 weeks. A transition zone was observed at 8 weeks, indicating the initiation of tissue generation. Histomorphologic analysis showed adipose and collagen connective tissue at 8 weeks. At 16 weeks, the relative composition was altered significantly. Adipose components occupied most of the tissue, and connective tissue was reduced. Blood vessels with endothelial lining were noted adjacent to adipocyte clusters, as well as in inter-adipocyte areas. The vessels had increased in number and were evenly distributed by 16 weeks. Our distraction technique produced more balanced adipose tissue generation than a non-distraction method, with co-development of adipose and vascular tissues.

## Introduction

Adipose tissue engineering methods have recently progressed to address the generation and development of fatty tissue to restore lost or defective sites. To treat large tissue defects, a tissue engineering strategy is necessary to provide promising results^1^. Autologous fat grafts have advantages for reconstruction, enabling augmentation of soft-tissue volume and contour defect correction. Autologous fat is biocompatible, available in sufficient amount in most patients, and naturally integrates with host tissues. However, adipose tissue transfer success is frequently limited because of its low and unpredictable graft survival rate^2^. Vascularization is the major limitation affecting the survival of grafted or engineered adipose tissue constructs^3, 4^. Viable vessels adjacent to the adipose tissue are crucial for nutrient diffusion and volume maintenance^5^. In addition, extracellular matrix elements secreted by endothelial cells greatly affect preadipocyte proliferation and differentiation^6^. There is significant evidence of co-regulation and interdependence between adipose tissue and the vasculature that supports it^7^.

Distraction techniques are utilized in various clinical fields, including distraction osteogenesis for craniosynostosis or hemifacial microsomia and soft-tissue distraction to improve chronic flexion contractures of digits^8, 9^. These techniques induce gradual histomorphogenesis and effective tissue generation secondary to distraction angiogenesis^10^. We postulated that the advantages of distraction techniques could improve adipose tissue generation.

The objective of this study was to evaluate the mechanical and biological effects of a distraction technique on adipose tissue generation. With the hypothesis that fat flaps gradually receding from each other can develop an adipose tissue construct, perforated polycarbonate syringe-shaped chambers were implanted in a rabbit model. This hypothesis was based on studies showing that fat flaps enclosed in perforated chambers induce adipose tissue formation and expansion^1, 5^. Two fat flaps elevated from the dorsal cervical area were enclosed in the chamber. After differing distraction periods, the generated tissue was harvested for morphologic and histologic analyses.

## Results

### Gross Morphology and Volume Analysis

**Figure 1:**
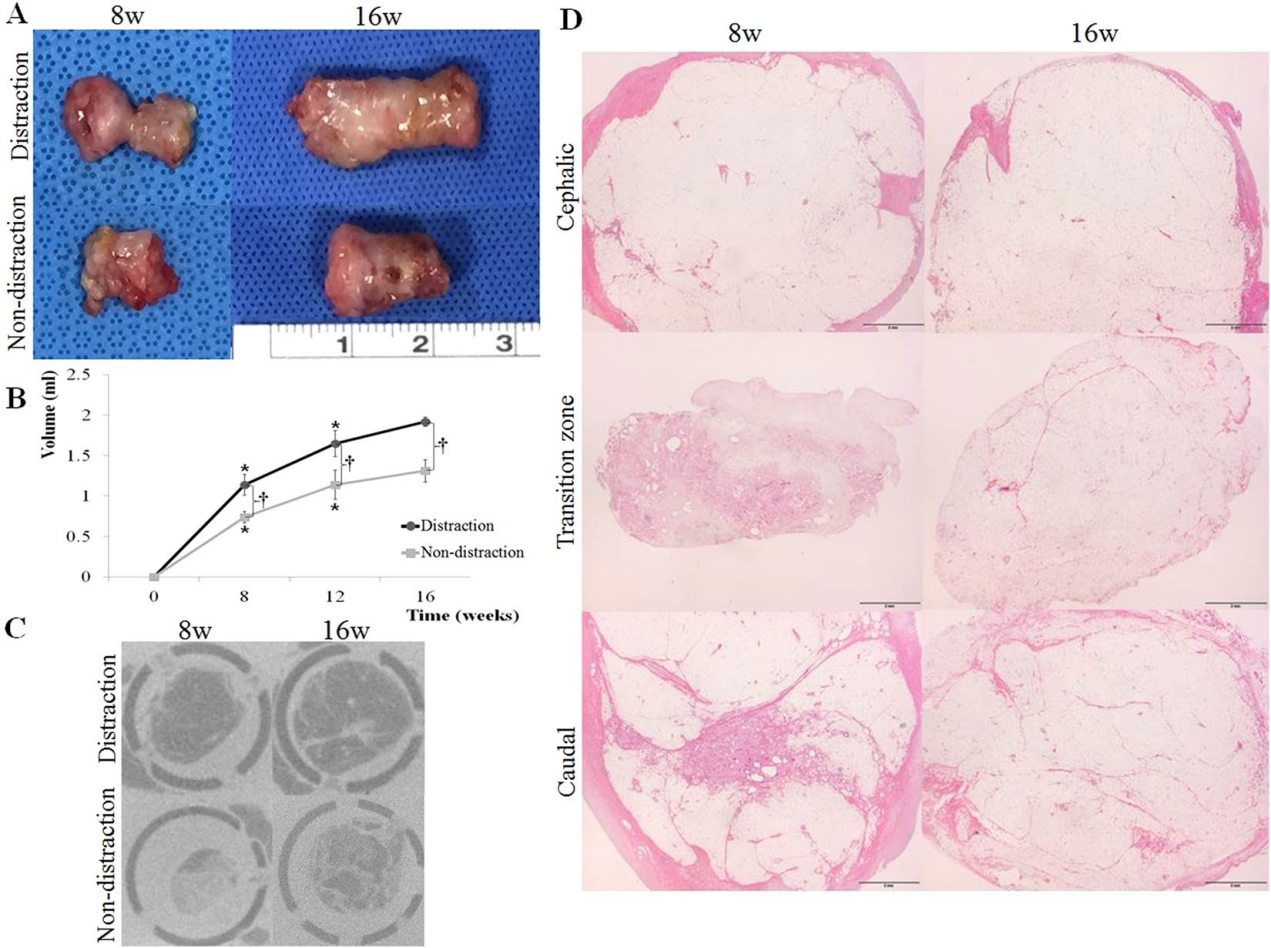
Gross observation (**A**), volume measurement (**B**), micro-computed tomography (CT) imaging (**C**), and cephalo-caudal microscopic observation (**D**) of the generated tissue in each group. In the distraction group, adipose tissue showed irregular contours, with a constricted transition zone, at 8 weeks. After 12 weeks of consolidation, adipose tissue construction was noted following volume expansion. In the non-distraction group the generated tissue exhibited pliable characteristics, with irregular contours, at 8 weeks. After 16 weeks, the adipose tissue had gained volume and fitted to the walls of the chamber (**A**). In the distraction group, the generated tissue volume gradually increased up to 16 weeks (final volume 1.92 ± 0.06 ml); nonetheless, significant expansion was noted at 8 and 12 weeks. The non-distraction group also exhibited a volumetric increment at 16 weeks (final volume 1.31 ± 0.14 ml), and significant increases were noted at 8 and 12 weeks. In comparative inter-group analysis, the distraction group volume was greater than that of the non-distraction group, indicating significant differences between groups at each time period (8, 12, and 16 weeks) (**B**). In micro-CT imaging, the distraction group exhibited adipose tissue formation in the central core at 8 weeks. At 16 weeks, enlarged adipose tissue occupied the chamber, with minimal connective tissue. Tissue generation was also noted in the non-distraction group, although the area was smaller compared with the distraction group. Tissue expansion had progressed at 16 weeks, but adipose and fibrous connective tissues were mixed (**C**). In histomorphologic analysis of the distraction group under low magnification (×10.25), the transition zone was small in diameter and exhibited inflammation at 8 weeks. Nonetheless, the area had enlarged and the inflammatory reaction had subsided at 16 weeks. The caudal area, adjacent to the caudally-based fat flaps, showed a connective tissue core at 8 weeks. Later observation at 16 weeks, nonetheless, exhibited adipose tissue components with septa (**D**). *p < 0.05, compared with measurement at the previous time point. ^†^p < 0.05, compared with the non-distraction control group.

Each group started with 15 animals (n = 15, distraction group; n = 15, non-distraction group). One rabbit in the distraction group showed delayed wound healing, and postoperative oozing necessitated additional conservative management. The other implantation sites exhibited no gross indication of acute inflammation or any abnormality in the chamber tissues. Gross observation of each group demonstrated gradual adipose tissue expansion. In the distraction group, adipose tissue exhibited irregular contours with a constricted transition zone at 8 weeks. The transition zone represented a core area where tissue was gradually generated. After 12 weeks of consolidation, adipose tissue exhibited regular contours and filled the perforated chamber. In the non-distraction group, generated tissue showed irregular contours and was pliable and fragile at 8 weeks. After maturation for 16 weeks, the adipose tissue had gained volume, and the surface fitted to the walls of the chamber. The non-distraction group tissue had a coarse capsular layer with heterogeneous color compared to the distraction group (Fig. 1A).

Harvested adipose tissue was subjected to volume measurement using the saline solution overflow method. In the distraction group, the fat pad volume gradually increased up to 16 weeks (final volume 1.92 ± 0.06 ml). Nonetheless, significant increases were noted at 8 weeks (1.14 ± 0.13 ml) and 12 weeks (1.65 ± 0.16 ml). The non-distraction group also exhibited a volumetric increment at 16 weeks (final volume 1.31 ± 0.14 ml), as well as significant growth at 8 weeks (0.74 ± 0.07 ml) and 12 weeks (1.14 ± 0.18 ml). In comparative inter-group analysis, the fat pad volume of the distraction group was greater than that of the non-distraction group, indicating significant discrepancies between groups at each time period (8, 12, and 16 weeks; p < 0.05) (Fig. 1B).

### Micro-Computed Tomography Imaging

Micro-computed tomography (CT) of samples, including the whole chamber, was performed immediately after tissue harvest. This enabled adequate evaluation before separating the tissue from the chamber. Images demonstrated generated tissues composed of adipose and connective tissues with distinguishable Hounsfield units (HUs) (fat, −100 to −50 HU; connective tissue, + 100 to + 300 HU).

The distraction group exhibited adipose tissue formation in the central area of the chamber at 8 weeks, when consolidation had been allowed for 4 weeks. At 16 weeks, more adipose tissue was noted, occupying most of the chamber space. In addition, there was minimal connective tissue. In the non-distraction group, mixed characteristic tissue with loose adipose and dense connective tissue components were present at 8 weeks. The generated tissue area was smaller than that seen in the distraction group. Tissue generation had progressed at 16 weeks; however, the connective tissue was dispersed, mimicking lipodystrophy (Fig. 1C).

### Histomorphologic Assessment

Histomorphologic analysis was performed using hematoxylin and eosin (H&E) and Masson’s trichrome staining methods. Serial tissue sections at three different sites (the cephalic, transition zone, and caudal areas) were obtained, followed by microscopic observation under low magnification (×10.25). The cephalic area consisted of cervical fat tissue originally and did not exhibit significant differences between 8 and 16 weeks. The transition zone, composed of newly-generated tissue, was noted to have a small diameter, with an inflammatory reaction at 8 weeks. By 16 weeks, however, the diameter had enlarged and inflammation had subsided. The caudal area, which was close to the caudally-based fat flaps, exhibited a connective tissue core at 8 weeks. At 16 weeks, however, adipose tissue components with septa were observed, which had undergone alteration in tissue characteristics (Fig. 1D). In contrast, the non-distraction group did not show transition zone and cephalo-caudal discrepancy.

**Figure 2:**
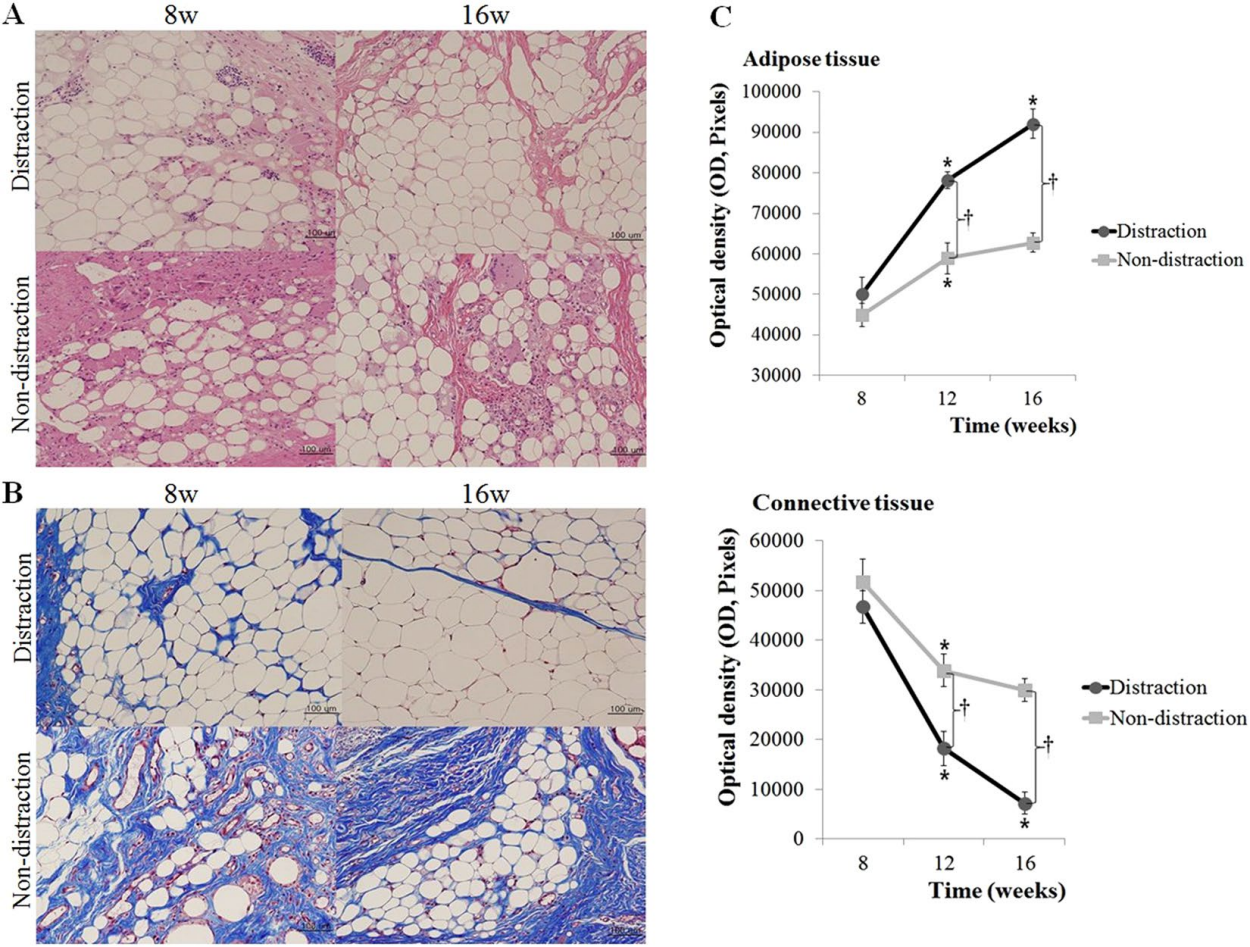
Histomorphologic evaluation. The distraction group exhibited uniform adipocytes at 8 weeks, as well as some connective tissue. At 16 weeks, the adipocytes consistently exhibited mature and viable characteristics. In the non-distraction group, connective tissue with inflammatory cell infiltration was prominent at 8 weeks. The images taken at 16 weeks showed notable fibrous tissue (**A**,**B**). In the distraction group, adipose and connective tissues gradually increased and decreased over 16 weeks, respectively. The same trends were noted in the control group up to 12 weeks. The distraction group exhibited higher adipose tissue quantity than the control group at 12 and 16 weeks, and connective tissue density showed an inverse relationship (p < 0.05). *p < 0.05, compared with measurement at the previous time point. ^†^p < 0.05, compared with the non-distraction control group.

Under high magnification (×200), generated tissue in the distraction group consisted of uniform adipocytes at 8 weeks, although connective tissue, including collagen and fibrin, was also observed. At 16 weeks, the relative composition was significantly altered. The adipose component occupied most of the tissue, and the connective tissue was reduced. Each adipocyte exhibited mature, viable characteristics, with no signs of atrophy, hypertrophy, or necrosis. In the control group, connective tissue was more prevalent than adipose tissue at 8 weeks, and inflammatory cell infiltration was noted. Subsequent results showed more regular adipocyte distribution at 16 weeks, but fibrotic connective tissue was still present (Fig. 2A,B).

Masson’s trichrome stain enabled tissue identification, and adipose and adjacent connective tissue were quantitatively evaluated. In the distraction group, adipose tissue increased significantly throughout 16 weeks (8 weeks: 50030 ± 4237 optical density [OD]; 16 weeks: 92104 ± 3636 OD). Adjacent connective tissue, however, exhibited a trend towards a gradual decrease (8 weeks: 46738 ± 3252 OD; 16 weeks: 7208 ± 2219 OD). In the non-distraction group, adipose tissue increased up to 12 weeks (8 weeks: 44939 ± 2817 OD; 12 weeks: 58894 ± 3841 OD), while adjacent connective tissue decreased (8 weeks: 51786 ± 4641 OD; 12 weeks: 33929 ± 3312 OD). At 16 weeks, the non-distraction group showed similar tendencies; however, the values were not distinguishable from the 12-week measurements. With regard to inter-group analysis, the distraction group exhibited a higher quantity of adipose tissue than the control group at 12 and 16 weeks, and an inverse relationship was noted in connective tissue density (p < 0.05) (Fig. 2C).

### Angiogenesis

**Figure 3:**
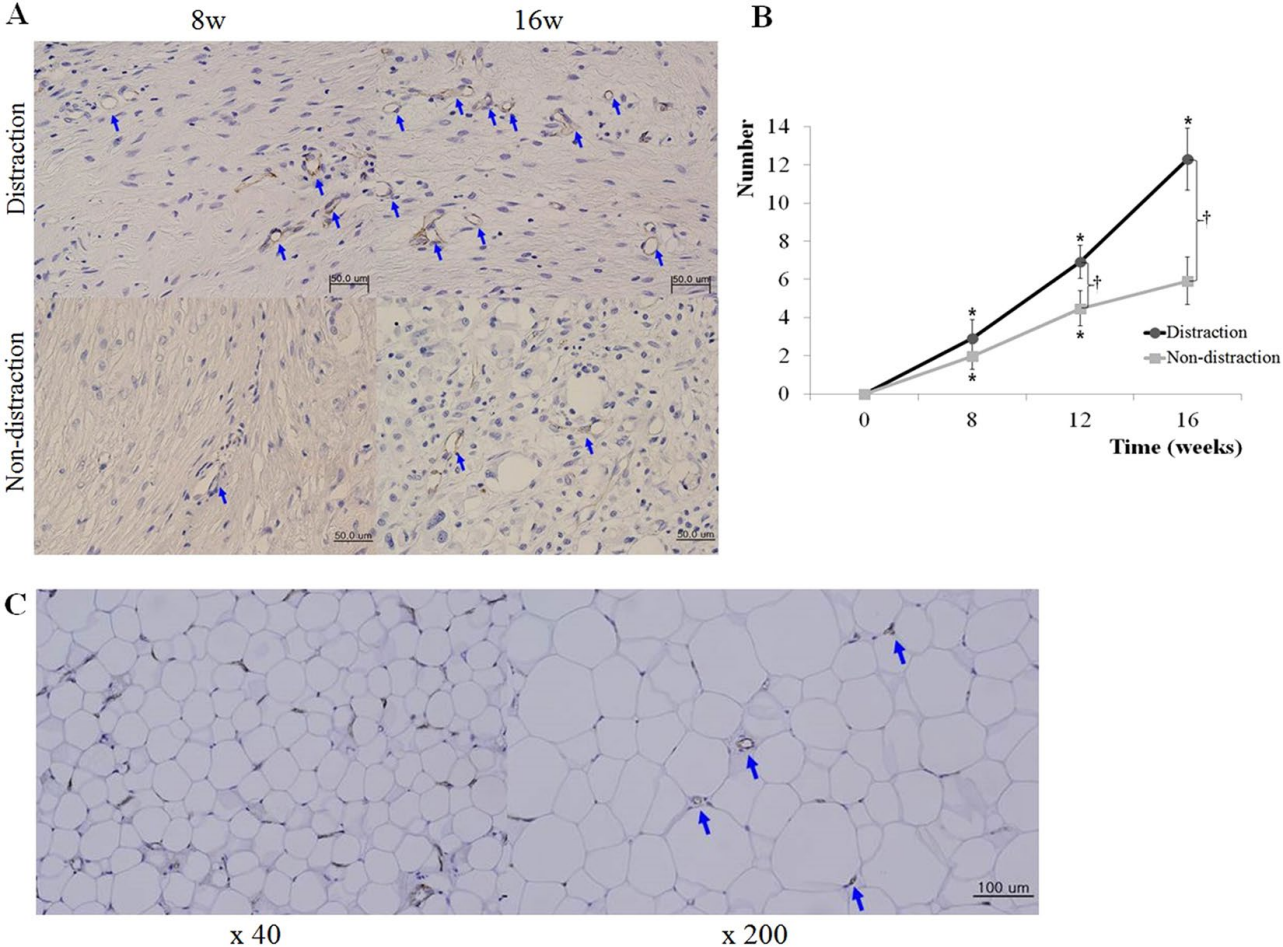
CD31 immunohistochemistry results showing evidence of angiogenesis. The arrows indicate endothelial lining with CD31-positive cells. Blood vessels were noted adjacent to adipocyte clusters at 8 weeks in the distraction group. The vessels increased in number and were evenly distributed at 16 weeks. In the non-distraction group, labeled cells were infrequent at 8 weeks. At 16 weeks, vessels were more apparent, but evidence of angiogenesis was less prominent than the distraction group (**A**). New blood vessels increased throughout the 16 weeks in the distraction group, and significant changes were noted at 8, 12, and 16 weeks. The non-distraction group exhibited increases up to 12 weeks. Comparative analysis revealed significant differences between groups at 12 and 16 weeks (p < 0.05) (**B**). In adipocyte clusters, the labeled capillaries were distributed throughout the uniform adipocytes. New blood vessels were noted among the adipocytes under high magnification in the distraction group, with prominent evidence of angiogenesis (**C**). *p < 0.05, compared with measurement at the previous time point. ^†^p < 0.05, compared with the control group.

CD31 immunohistochemistry was performed to assess angiogenesis. Blood vessels with endothelial lining were noted at the junction between the adipose and connective tissues in the distraction group. The vessels were increased in number and evenly distributed at 16 weeks, compared with earlier time points. Evidence of angiogenesis was less prominent in the non-distraction group (Fig. 3A).

New blood vessels increased throughout the 16 weeks in the distraction group. Significant changes were noted at 8, 12, and 16 weeks (8 weeks: 2.92 ± 0.98; 16 weeks: 12.3 ± 1.62). The non-distraction group also had more blood vessels up to 12 weeks, showing significant increases at 8 and 12 weeks (8 weeks: 1.96 ± 0.67; 12 weeks: 4.48 ± 0.91). In inter-group comparative analysis, the numbers of blood vessels were significantly different between groups at 12 and 16 weeks (p < 0.05) (Fig. 3B). Furthermore, new blood vessels were noted among adipocytes at high magnification (×200) in the distraction group, and evidence of angiogenesis in adipocyte clusters was more prominent than in the non-distraction group. Inter-adipocyte angiogenesis was a significant finding in the distraction group (Fig. 3C).

## Discussion

Adipose tissue is a metabolically active and highly vascularized organ. It has been suggested that every adipocyte has one or more supportive capillaries^3^. In addition to supplying oxygen and nutrients, the vascular system is essential for transporting cytokines, hormones, and growth factors. The vasculature, therefore, has a substantial role in adipocyte survival and maintenance^4^. An adipose tissue construct exceeding a critical size may suffer from poor supply and subsequent cell necrosis^7^. In the clinical setting, transferred adipose tissue is at risk of volume loss of up to 60%^1^. Adipocyte necrosis results from an inadequate initial blood supply and low tolerance to ischemia. A number of approaches have been developed to improve adipose tissue constructs, but the desired characteristics of cellular components in these constructs have remained unclear.

Findlay *et al*. developed a perforated chamber that was subcutaneously inserted into the swine groin enclosing a fat flap based on the superficial circumflex iliac pedicle^5^. They reported new vascularized tissue that filled a 78.5-ml chamber in 22 weeks. Histologic analysis demonstrated increased adipose tissue volume associated with adipocyte proliferation.

Tissue engineering chambers (TECs) have been utilized to generate adipose tissue constructs^1, 5, 11–13^. Peng *et al*. noted that collagen tissue was dominant during early observations (at 30 days), but it was replaced by newly-formed adipocytes over time^11^. They suggested four stages of TEC adipose tissue generation: inflammation period (0–15 days), angiogenesis stage (15–30 days), adipogenesis stage (30–45 days), and maturation stage (45–60 days). In the inflammation stage, surgical procedures and chamber implantation prompted macrophage and mesenchymal stem cell (MSC) infiltration, followed by the release of angiogenic and growth factors. The inflammatory reaction remarkably enhanced angiogenesis, extracellular matrix (ECM) expansion, and perivascular cell proliferation. In the adipogenesis stage, perivascular cells switched into adipose precursor cells, and adipogenic differentiation occurred if there was an adequate blood supply. Finally, in the maturation stage, adipose tissue regeneration was completed following the expansion of newly-generated adipocyte tissue and ECM remodeling.

We hypothesized that a TEC using the fat flap distraction technique could induce gradual generation and maintenance of adipose tissue with natural histologic characteristics. Early observation at 8 weeks demonstrated a constricted transition zone, which confirmed gradual tissue generation from the core area. At 16 weeks, the tissue exhibited regular contours and filled the chamber. The distraction method achieved more efficient volume expansion than that observed in the non-distraction group, where the fat tissues had been separated by a distance since chamber implantation (Fig. 1A).

The distraction technique has been utilized in reconstructive procedures for which 1) gradual tissue generation is advantageous, 2) a considerable amount of tissue is required, or 3) adjacent tissue quality is relatively poor because of hypoxia, irradiation, or chronic inflammation^8, 14–17^. Tissue generation using distraction involves mechanical traction, transient ischemia, and subsequent angiogenesis^10^. The generated tissues have demonstrated stability and resistance against poor vascularization^18, 19^. Notably, our distraction group exhibited mature adipocytes with significant vascularity in histomorphologic and quantitative analyses. In addition, the generated tissue at 8 weeks consisted of connective tissue in the core area, but this had been replaced with mature adipose tissue at 16 weeks, which is in accordance with previous findings (Fig. 1D)^1, 11, 12^.

The synergy between angiogenesis and adipogenesis has been verified through the incorporation of preexisting vascularized adipose tissue^4, 6^. Changes in the mechanical force within the chamber provide additional mitogenic stimulus in accordance with mechanotransduction^20^. Adequate mechanical force is crucial in adipogenesis with regard to mechanobiology, since native stiffness of a culture substrate (2 kPa) can induce adipogenesis^21^. Softer or stiffer substrates have led to neurogenic or myogenic differentiation, respectively^22, 23^. These effects are mediated by altered arrangement of the cytoskeleton^24^. Conversely, a lack of adequate cell-to-cell contact in fat flaps has been associated with a poor adipogenesis rate^25^. Our histomorphologic and quantitative findings demonstrate comparable results in terms of vascular and adipose tissue co-development (Figs 2 and 3). In our distraction group, vascular growth among adipocytes supported the viability of the adipose tissue construct (Fig. 3). Meanwhile, non-distraction group exhibited a fibrous connective tissue construct without adequate tissue contact and mechanotransduction.

Nie *et al*. suggested delivering adipose-derived stem cells (ASCs) through an acellular dermal matrix, and several authors have successfully developed a model in different types of scaffolds^26^. In our distraction model, connective tissue initially generated at the transition zone expanded into adipose tissue exhibiting fullness and natural contours. The initial tissue could promote adipose tissue formation by serving as a natural adipogenic scaffold.

Aseptic inflammation has been reported to promote adipose tissue regeneration^11^. Lilija *et al*. found that adipogenesis was induced by macrophage-derived factors, such as monocyte chemotactic protein-1 (MCP-1), which recruited macrophages, and bone marrow-derived precursor cells^12^. Adipogenesis in TECs is thought to optimize the relationship between controllable inflammation and adequate adipogenesis. Inflammation is usually considered a common factor connecting various comorbidities, but a recent study noted that hypoxia could be a potential risk factor for chronic inflammation^27^. Hypoxia can lead to inflammation in adipose tissue and induce gene expression in macrophages and adipocytes. It is believed that local inflammation related to hypoxia serves as a physiological promoter for angiogenesis and ECM remodeling in adipose tissue; however, when inflammatory reactions persist, chronic inflammation occurs^28^. During chronic inflammation, vascular networks cannot provide adequate oxygen to adipocytes, and local hypoxia arises, which could trigger adipose tissue dysfunction^29^. In that instance, hypoxia occurs in a group of adipocytes distal to the vascular network as adipose tissues expand^30^. The researches regarding impaired adipogenic capacity in hypoxic condition elucidate distinguishable results in our distraction and non-distraction groups. When hypoxic and inflammatory states were overcome by sufficient vascularity with an adequate matrix, mature adipose tissue could be generated in the distraction group. In contrast, the void in non-distraction group was filled with fibrous connective tissue, infiltrated with inflammatory cells. Although there was an adipose component, evidence of chronic inflammation still existed.

Lilja *et al*. suggested that gene expression and various cytokines are involved in the early development of engineered adipose tissue^12^. They utilized TECs in a mouse model and reported macrophages entering the TEC at 12 hours post-insertion. This caused prompt increments in MCP-1 and macrophage inflammatory protein-1 alpha (MIP-1α), providing a signal for further macrophage recruitment. The cytokines attracted precursor cells, including MSCs, to the chamber to facilitate tissue generation. The authors reported that cytokine levels decreased in the chamber beginning at 2 days post-insertion, whereas growth factors such as tumor necrosis factor-α, lipocalin-2, and interleukin-1β increased. These factors served as additional stimuli for *de novo* blood vessel formation. In addition, different stages of angiogenesis are modulated by ASCs and adipocytes. A recent study reported that vascular endothelial growth factor (VEGF) expression by ASCs and adipocytes could destabilize existing vessels for vascular sprouting^7^. Following destabilization, endothelial cell migration and proliferation are induced by angiotensin II and basic fibroblast growth factor (b-FGF) released from cellular components of adipose tissue clusters^31, 32^. Adipocyte-derived factors, including leptin, monobutyrin, and adenosine, enhance the process and promote vascular fenestration^33, 34^. Several important mediators also play roles in adipogenesis-angiogenesis crosstalk. Peroxisome proliferator-activated receptor gamma (PPAR-γ) and the human CCAAT/enhancer binding protein alpha (C/EBP-α), the main modulators of adipogenesis, are activated through various endocrine modes, and VEGF expression in adipocytes utilizes common mediators^7, 35, 36^. The linkage between adipocyte- and endothelial cell-derived mediators suggests interdependence between the two components.

**Figure 4:**
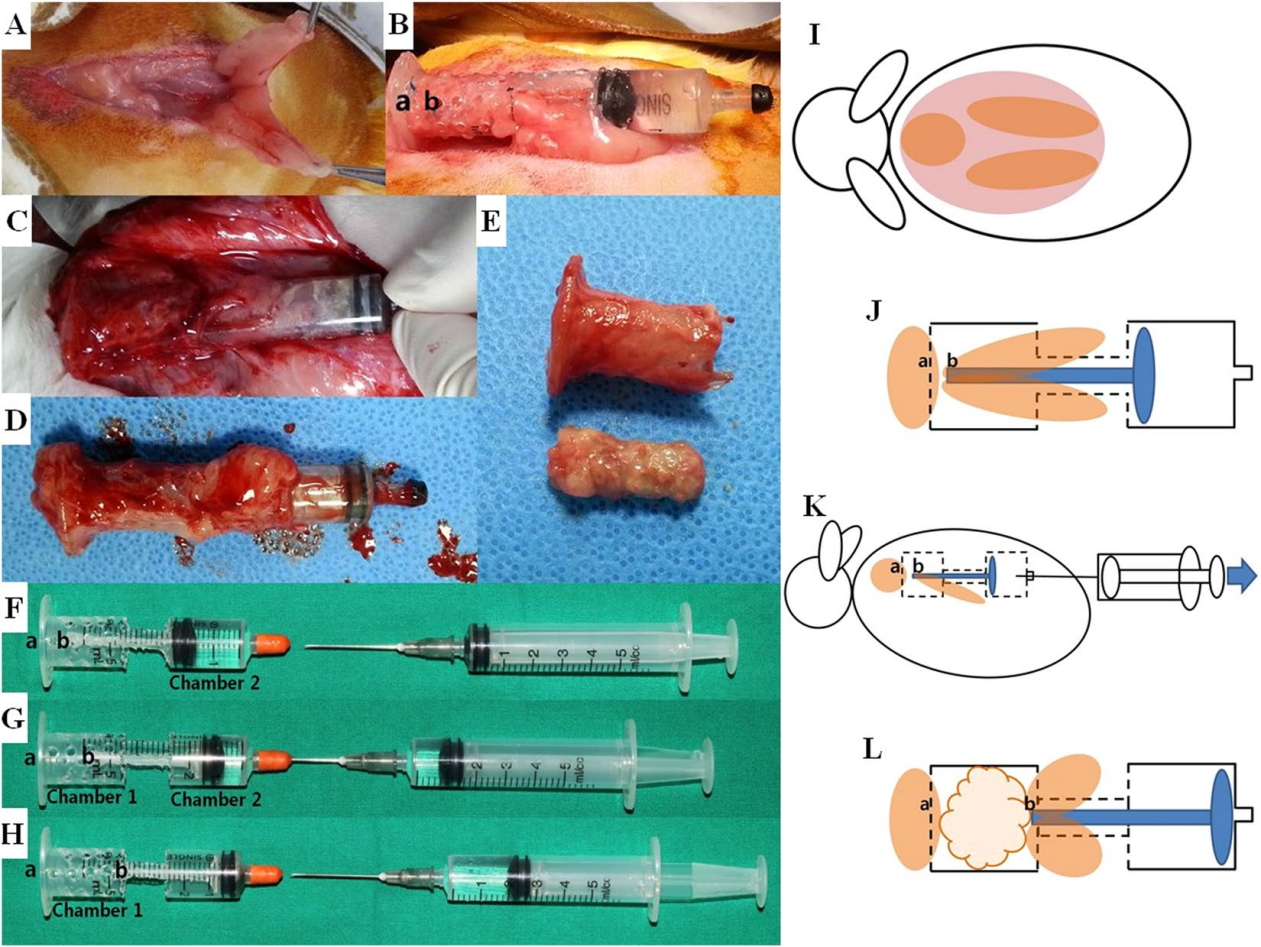
Adipose tissue formation using a distraction technique. Two fat flaps are elevated on the dorsum of the cervical area (**A**), inserted into the chamber, and the tips of the flaps are placed at point (b) for dynamic distraction. A counter fat tissue is sutured at point (a), the fixed part of the chamber (**B**). Distraction is performed for an assigned period, and the chamber with adjacent tissue is harvested (**C**,**D**). Newly-generated adipose tissue is carefully separated from the chamber (**E**). In the distraction chamber model, fat tissues fixed at points (a) and (b) points are in contact with each other (**F**). Distraction is performed through gradual extraction of saline solution from chamber 2 (**G**). As point (b) recedes from point (a), chamber 1 expands (**H**). Schematically, two fat flaps are elevated following subcutaneous dissection (**I**). The flaps are inserted into the chamber, and the tips of flaps are placed at point (b) for dynamic distraction. A counter fat tissue is sutured at point (a), the fixed part (**J**). The entire distraction chamber is inset in the subcutaneous layer, and distraction is performed using a syringe (**K**). Newly-generated tissue can be observed between points (a) and (b) after a consolidation period (**L**).

External volume expansion (EVE) methods have been studied to verify their regenerative effects on adipose tissue. A number of outcomes have been reported, and the results varied depending on the experimental protocols^37–40^. Negative pressure and duration of application could affect the resulting tissue assessments. A recent study using a porcine model demonstrated the vascular remodeling and maturation effects of EVE; however, proliferation of adipocytes and keratinocytes was limited^41^. Our chamber model differed from EVE, since the tissues were not subjected to negative pressure. They were generated in perforated domains, which were covered by adipose tissue. Enlargement of the perforated chamber (chamber 1) was achieved by reducing the counter-chamber (chamber 2), not by negative pressure (Fig. 4). Consequently, the generated tissues were not subjected to the stresses of negative pressure or recoiling forces. A comparative analysis would clarify the histomorphologic differences in the involved tissues between EVE methods and our perforated chamber using distraction technique.

A limitation of our study was that the generated tissue was restricted by the perforated chamber throughout the experimental period. An additional consolidation period following chamber explantation could allow further analysis of the tissue, which would be left under the direct influence of adjacent tissues. Findlay *et al*. reported long-term stability of adipose tissue generated from a vascularized fat flap up to 5 months after chamber removal^5^, and tissue-engineered adipose flaps using TECs have shown persistence^1, 42^. Further research using TECs combined with distraction may reveal detailed characteristics.

Previous publications have utilized various terminology, including adipose tissue generation^1, 43^, regeneration^11, 13^, development^12, 44^, and expansion^45, 46^. To accurately define tissue regeneration, it is important to ensure regenerative processes of the tissue that is injured or undergoing degenerative changes. In addition, the origin of adipocytes needs to be clarified, with *de novo* adipogenesis of particular interest. We utilized the terms adipose tissue generation and formation. Nonetheless, tracking cell sources is important in future studies, and it is critical to state whether they have migrated or been recruited or generated.

Our results revealed a significant correlation between adipose tissue formation and angiogenesis induced by a distraction technique. An adjacent connective tissue network supported tissue generation, acting as a natural scaffold. The non-distraction model resulted in adipose and fibrous tissue formation in the perforated chambers, whereas the distraction method generated well-vascularized adipose tissue with more balanced adipocyte-endothelial interaction. Further research utilizing larger distraction chambers with long-term observation should precede clinical applications.

## Methods

### Experimental Animals

This study protocol was conducted under the regulations of the Review Board of Experimental Ethics, College of Medicine, Yonsei University, Seoul, Korea. The Institutional Animal Care and Use Committee (IACUC) approval number was 2014-0326. All animals were maintained, fed, and euthanized in accordance with standard protocols of the Department of Laboratory Animal Resources, Yonsei Biomedical Research Institute. Thirty male New Zealand albino rabbits (2500–3500 g) were selected for assignment to three experimental and three control groups.

### Experimental Study

**Figure 5:**
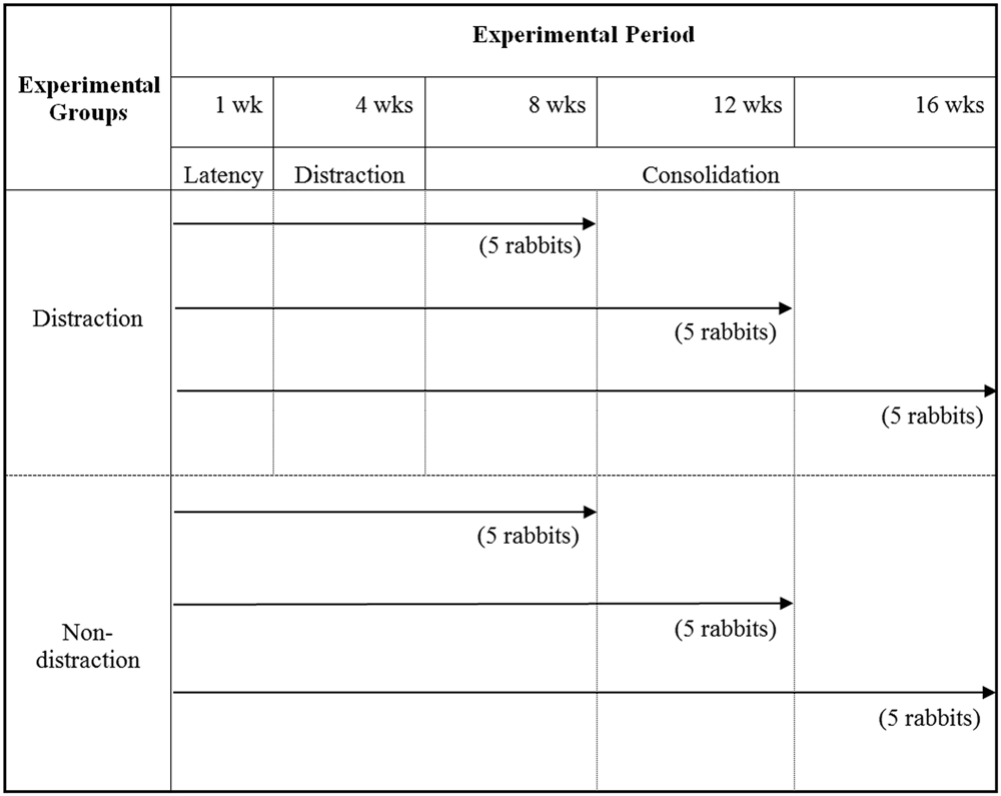
Experimental time table. After 7 days of latency following surgery, distraction was conducted at the rate of 0.7 ml/7 days × 3 times (total of 2.1 ml over 21 days). Ten rabbits (5 in the distraction group and 5 in the non-distraction group) were euthanized at postoperative 8, 12, and 16 weeks. Distraction and non-distraction subgroups were assigned according to the consolidation period.

New Zealand albino rabbits were used for the adipose tissue distraction model. Surgery was performed after an overnight fast. Animals were anesthetized intramuscularly with Zoletil 50 (Virbac, France) and 2% Rompun solution (Bayer, Leverkusen, Germany) (1:2 ratio, 1 ml/kg). Dorsal surfaces of the cervical area were shaved and disinfected with povidone-iodine solution. A 7-cm vertical incision was made in the cutaneous layer of the prepared area. For the experimental group, two fat flaps measuring approximately 1 × 1 × 5 cm^3^ were elevated after subcutaneous dissection around the cervical area. The researcher-manufactured perforated polycarbonate syringe-shaped chambers were implanted within the subcutaneous layer, and fat flaps were fixed at (a) and (b) points with #4-0 nylon sutures (Fig. 4B,F,J). Subcutaneous and skin layers were repaired with #3-0 vicryl and #4-0 nylon sutures, respectively. Afterwards, 5 mg/kg enrofloxacin (Baytril®, Bayer Healthcare, Shawnee Mission, KS, USA) was administered intramuscularly for 3 days to prevent infection. After 7 days of rest, distraction in the distraction group was conducted at a rate of 0.7 ml/7 days × 3 times (total 2.1 ml over 21 days) (Fig. 4F–H). Consolidation periods were 4, 8, and 12 weeks (5 rabbits × 3 subgroups). For the non-distraction group, a perforated polycarbonate chamber was implanted in a state of complete distraction, and cervical fat tissues were fixed at each end of the implant (Fig. 4H). Ten rabbits were euthanized at postoperative 8, 12, and 16 weeks, and the generated tissue between points (a) and (b) points, plus the adjacent fat lumps, were harvested for analysis (Figs 4 and 5).

### Quantitative and Histologic Analysis

Noninvasive quantification of adipose tissue was performed using micro-CT on the day of tissue harvest. The raw file was reconstructed into an ordered sequence of two-dimensional (2D) sections of the scan region. The final grayscale file reflected the apparent density of each voxel, with denser and less dense tissues appearing brighter and darker, respectively. *In vivo* micro-CT scans have been validated over a broad range of body weights and adiposity^47^.

Newly-generated and adjacent normal fat tissues were subsequently harvested. The fat pad was dissected, and its volume was measured using the liquid overflow method. Following gross observation, tissue sectioning was performed in the middle of points (a) and (b), at the farthest site from the previously existing adipose tissue. Sections of the fat pad were stained with (1) H&E, (2) Masson’s trichrome, and (3) CD31 immunohistochemistry labeling methods prior to examination with light microscopy (BX61VS, Olympus Corp., Tokyo, Japan).

Masson’s trichrome staining was performed to analyze connective tissues, including collagen and fibrin. The staining solution was formulated using Bouin’s solution (picric acid solution, 75 ml; 37% formalin, 25 ml; glacial acetic acid, 5 ml); Weigert’s iron hematoxylin solution (hematoxylin, 1 g; 95% ethanol, 100 ml; ferric chloride, 2 g; concentrated HCl, 1 ml; distilled water, 95 ml); Biebrich scarlet-acid fuchsin solution (1% Biebrich scarlet, 90 ml; 1% acid fuchsin, 10 ml; glacial acetic acid, 1 ml); phosphomolybdic–phosphotungstic acid (phosphomolybdic acid, 2.5 g; phosphotungstic acid, 2.5 g); and an aniline blue solution (aniline blue, 2.5 g; distilled water, 100 ml; glacial acetic acid, 2 ml).

A semi-quantitative analysis of adipose and connective tissue densities was conducted using MetaMorph^®^ image analysis software (Universal Image Corporation, Buckinghamshire, UK). The results were reported as the average OD on five different digital images, and the mean was calculated for each group of animals. OD can quantify the opacity of slides exposed to transmitted light, and outcomes could be considered inversely proportional to the grayscale values, which are related to the amount of spectral light.

CD31 immunohistochemistry staining was performed to measure the quantitative extent of angiogenesis within the adipose tissue. Sections were pretreated with 3% hydrogen peroxide solution for 10 minutes to block endogenous peroxidase and then processed with protein block serum-free reagent (X0909; DAKO, Carpinteria, CA, USA) for 30 min to prevent nonspecific binding. The sections were incubated at 4 °C overnight with primary antibodies (rabbit anti-VEGF, RB-222-P, Laboratory Vision, Fremont, CA; anti-mouse platelet endothelial cell adhesion molecule-1 [PECAM/CD31] polyclonal antibody, M20, Santa Cruz Biotechnology, Santa Cruz, CA, USA), followed by incubation at room temperature for 20 min with DAKO Envision Kit (DAKO) secondary antibodies.

To evaluate neovascularization, we counted the number of CD31-positive vessels enclosed with a single layer of endothelial cells and without a muscular layer. A comparative analysis of the number of blood vessels was performed in each high-power field (×200). For each slide, the number of vessels was measured at five different sites at the junction between the adipose and connective tissues, where the vessels were abundant. Vascular density was presented for each time period in each group of animals.

### Statistical Analysis

Unpaired t-tests were performed to compare fat pad volumes, the quantity of generated adipose and connective tissues, and the number of new blood vessels between experimental and control groups. One-way analysis of variance was used to detect differences in these variables between time periods in each group. Significant analysis of variance results were followed by post hoc tests for pairwise comparisons, adjusted by the Bonferroni correction. P values < 0.05 were considered statistically significant.
